# SR calcium handling dysfunction, stress-response signaling pathways, and atrial fibrillation

**DOI:** 10.3389/fphys.2015.00046

**Published:** 2015-02-19

**Authors:** Xun Ai

**Affiliations:** Department of Cell and Molecular Physiology, Loyola University ChicagoMaywood, IL, USA

**Keywords:** atrial fibrillation, calcium handling, arrhythmogenesis, stress-response kinases, heart failure, aging

## Abstract

Atrial fibrillation (AF) is the most common sustained arrhythmia. It is associated with a markedly increased risk of premature death due to embolic stroke and also complicates co-existing cardiovascular diseases such as heart failure. The prevalence of AF increases dramatically with age, and aging has been shown to be an independent risk of AF. Due to an aging population in the world, a growing body of AF patients are suffering a diminished quality of life and causing an associated economic burden. However, effective pharmacologic treatments and prevention strategies are lacking due to a poor understanding of the molecular and electrophysiologic mechanisms of AF in the failing and/or aged heart. Recent studies suggest that altered atrial calcium handling contributes to the onset and maintenance of AF. Here we review the role of stress-response kinases and calcium handling dysfunction in AF genesis in the aged and failing heart.

## Introduction

Clinical studies have shown that atrial fibrillation (AF) is the most common cardiac arrhythmia and has an associated high risk of mortality and morbidity (such as stroke and heart failure) in the aging population (Benjamin et al., 1994; Psaty et al., 1997; Podrid, 1999; Go et al., 2001; Miyasaka et al., 2006; Rich, 2009). Both heart failure (HF) and aging have been shown to be independent risk factors for AF (Benjamin et al., 1994; Kannel et al., 1998; Ehrlich et al., 2002; Neuberger et al., 2007). HF affects nearly 15 million people worldwide (Cowie et al., 1997; Hershberger et al., 2003). One third to one half of patients with HF develop AF (Markides and Peters, 2002). New-onset AF among HF patients has consistently been associated with a 2-fold increase in all-cause mortality. Due to an aging population, the prevalence of both AF and HF is predicted to more than double by 2050 (Linne et al., 2000; Di Lenarda et al., 2003). The high prevalence of these multiple co-morbidities (Wang et al., 2003) (e.g., very frequent co-existence of HF, AF, with aging) has tremendous impact on the quality of life and daily functioning of elderly individuals, and is a significant financial burden worldwide (Linne et al., 2000; Di Lenarda et al., 2003). However, pharmacological treatment and prevention strategies remain ineffective due to the incomplete understanding of the underlying molecular and electrophysiologic mechanisms of AF genesis and development.

Accumulating evidence suggests that intrinsic stress (e.g., oxidative stress and chronic inflammatory stress) are markedly enhanced in aging, HF, and AF, while the aged and pathologically altered hearts have been shown to exhibit a higher susceptibility to extrinsic stress stimuli (Belmin et al., 1995; Beckman and Ames, 1998; Juhaszova et al., 2005; Neuman et al., 2007; He et al., 2011; Ismahil et al., 2014). The mitogen-activated protein kinase (MAPK) cascade is composed of a family of signaling cascades, which act as critical regulators of cell survival and growth in response to both intrinsic and extrinsic stress challenges. The three MAPK subfamilies c-Jun N-terminal kinase (JNK), extracellular signal-regulated kinases (ERKs), and p38 MAPKs have been the focus of extensive studies to uncover their roles in cardiac disease development (Davis, 2000; Karin and Gallagher, 2005; Ramos, 2008; Rose et al., 2010). The impacts of these stress-response kinases on sarcoplasmic reticulum (SR) calcium (Ca) handling proteins have begun to be revealed (Ho et al., 1998, 2001; Takahashi et al., 2004; Hagiwara et al., 2007; Scharf et al., 2013; Huang et al., 2014). Extensive studies suggest that alterations of Ca handling proteins including RyR2, phospholamban [PLB, an inhibitory protein of SR Ca pump (SERCA2)], and L-type Ca channels (Cav1.2) contribute to changed intracellular Ca transients and diastolic SR Ca release that in turn lead to Ca-triggered ventricular and atrial arrhythmogenesis (Schulman et al., 1992; Wu et al., 1999; DeSantiago et al., 2002). Thus, this review focuses on the recent progress in understanding the role of stress-response kinases and calcium signaling dysfunction in AF genesis in the aged and failing heart.

## Electrical remodeling precedes AF onset and development

**Figure 1:**
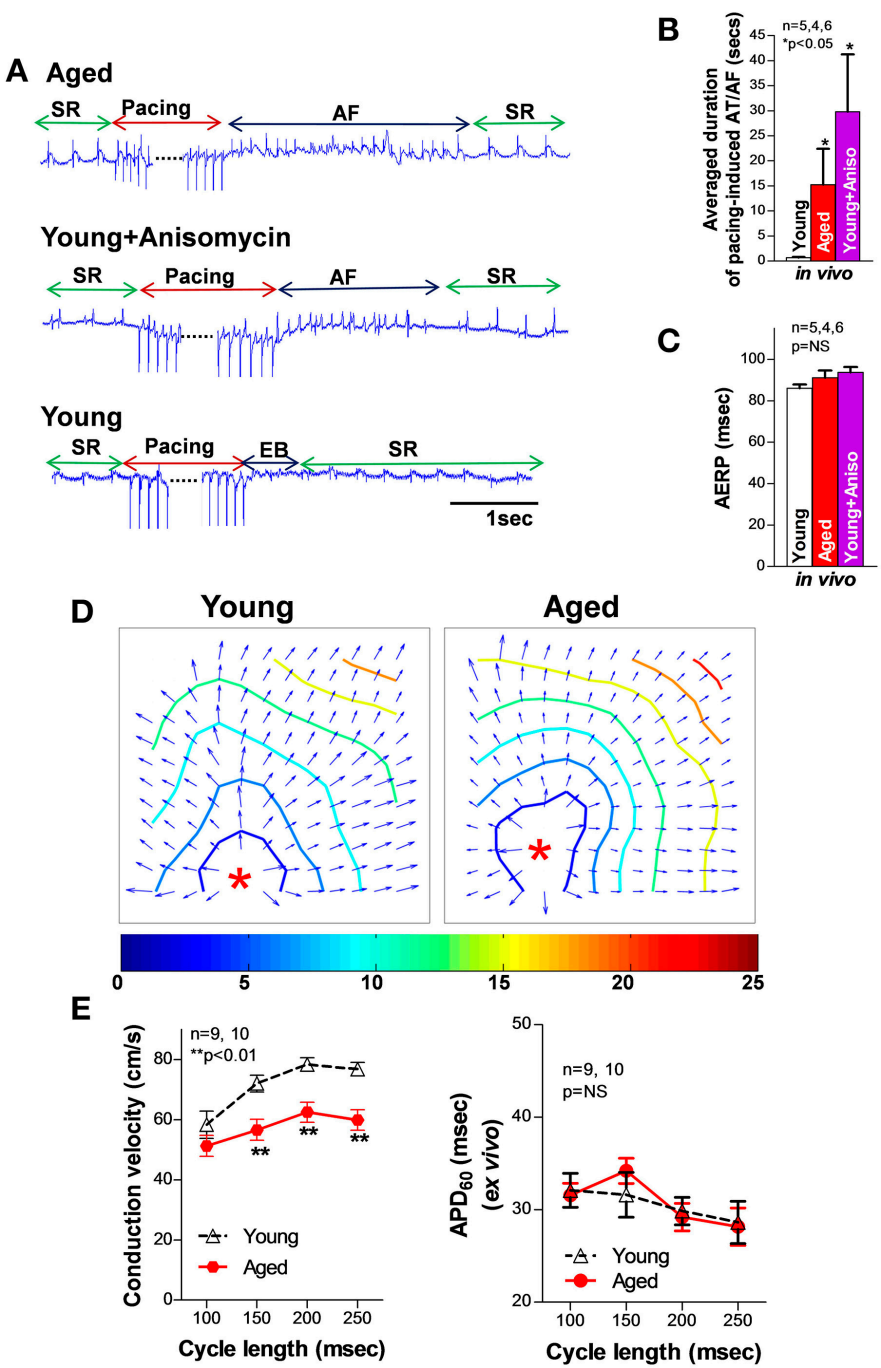
**(A)** Representative electrograms (EG) of burst pacing (for 30 s at 6× diastolic threshold, CL = ±5 ms of atrial effective refractory period (AERP) induced AF followed by self-reversion to sinus rhythm (SR) in open-chest aged (top row) and anisomycin-challenged young rabbit LA (middle row), and self-restored SR after burst pacing induced extra beats (EB) in sham control young rabbit LA (bottom row). **(B–C)** Summarized data of average duration of pacing-induced AT/AF as well as AERP in open-chest aged rabbit LA and young rabbit LA with or without anisomycin treatment (^*^p < 0.05 and *p* = NS, respectively). **(D)** Representative isochronal maps from young and aged rabbit hearts subjected to pacing at a CL of 200 ms (π indicates the pacing sites). **(E)** Summarized optical mapping CV data show that aged rabbits exhibited a CL-dependent, slower conduction but unchanged AERP vs. young controls. This figure is modified from Yan et al. (2013).

It is generally believed that abnormal triggers initiate AF, while an arrhythmogenic substrate sustains it (Nattel et al., 2008). While reentry circuits due to the formation of arrhythmogenic substrate including molecular and structural remodeling have been demonstrated to be important in AF development (Allessie et al., 1976; Mandapati et al., 2000), the underlying mechanisms of AF initiated by abnormal ectopic trigger activities remain unclear. Extensive studies in ventricular myocytes have shown that ectopic activities can occur by prolonged action potential duration (APD) causing early afterdepolarizations (EADs) and by spontaneous SR Ca releases leading to delayed afterdepolarizations (DADs) (Nattel et al., 2008). EADs normally occur with abnormal depolarization during phase 2 or phase 3 of the action potential (AP). While ventricular myocytes can only develop phase 2 EADs, atrial myocytes do not produce phase 2 EAD but may produce late phase 3 EADs with an abbreviation of the atrial APD (Burashnikov and Antzelevitch, 2003; Patterson et al., 2005). Studies suggest that electrical remodeling of atrial membrane ion channels (e.g., Ca and potassium channels) leads to altered APD and atrial effective refractory period (AERP); both have been found to be associated with the development of AF (Marx et al., 2000; Christ et al., 2004; Nattel et al., 2007). Before the onset of AF, shorter AERPs were associated with a higher inducibility of AF, while longer AERPs and slowing atrial conduction velocity, which may cause a pro-arrhythmogeinc shortening of the conduction wavelength, Rensma et al. (1988) were found to be linked to AF development in HF patients and animals (Huang et al., 2003; Sanders et al., 2003). In aged rabbit left atrium, we found that a slight reduction in AERP and unchanged action potential duration (APD_30_ and APD_60_; pacing cycle length = 200 ms) were associated with slowed conduction velocity and a markedly increased pacing induced AF compared to that of young controls (Figure 1)(Yan et al., 2013). Although similar results of slightly altered APD and AERP were also reported in aged canine and rat atria, Anyukhovsky et al. (2005) and Huang et al. (2006) studies from coronary artery bypass graft (CABG) surgery patients suggest that AERP was positively correlated with age (Sakabe et al., 2003). However, the molecular and electrophysiological properties of human hearts are known to be varied and complicated, especially when co-existing pathological conditions (such as HF or myocardial infarction) are present. While these results need to be further confirmed in healthy aging human donor hearts and further validated in other animal aging models, studies suggest that atrial electrical remodeling was found to occur long before the first occurrence of AF, and was not always correlated with the occurrence of sustained AF in patients and animal models (van der Velden et al., 2000; Kanagaratnam et al., 2008). In addition, late-phase 3 EADs have only been shown to be responsible for the immediate initiation of AF following termination of paroxysmal AF, but not in the case of newly onset AF or reoccurrence of AF that has been terminated for a long time (Timmermans et al., 1998; Oral et al., 2003). Thus, other features of the arrhythmogenic substrate such as SR Ca handling dysfunction, a generally acknowledged arrhythmogenic factor of generating DADs, could play an important role in failing or age-related enhancement of atrial arrhythmogenicity.

## Atrial SR Ca handling in AF genesis

Although Ca handling in atrial myocytes is similar to that of ventricular myocytes, there are some important structural and cellular signal differences between atrial and ventricular myocytes. Atrial myocytes are thinner and longer, Walden et al. (2009) which may lead to a longer delay between APs and Ca transients at the center of the cells. This property of the atrial cell can increase the instability of Ca propagation, which is pro-arrhythmogenic. In addition, atrial myocytes exhibit a different Transverse tubules (T-tubules) structure compared to ventricular myocytes. T-tubules are an important sub-cellular network involved in SR Ca dynamics in ventricular myocytes (Wang et al., 2001; Brette and Orchard, 2003; Franzini-Armstrong et al., 2005; Ibrahim et al., 2010). T-tubules are located at the z-line of the myocyte and provide close coupling of L-type Ca channels to ryanodine receptors (RyRs) on the SR membrane. This structure allows rapid intracellular Ca triggered SR Ca release in response to electrical excitation (Franzini-Armstrong et al., 2005). Emerging evidence suggests that an atrial T-tubule network is present in large mammalian species including humans, sheep, dogs, cows, and horses (Dibb et al., 2009; Lenaerts et al., 2009; Wakili et al., 2010; Richards et al., 2011) although atrial T-tubular networks are less abundant and less organized compared to that in the ventricles. While it was previously believed that atrial T-tubules were virtually absent in the small rodents,(Forbes et al., 1990; Berlin, 1995) a recent report by Frisk et al. (2014) showed similar structural organization and density of the T-tubules in pig and rat atria. A disorganized T-tubule network has been found to contribute to SR Ca release dysfunction in failing ventricular myocytes from both human and HF animal models (Balijepalli et al., 2003; Louch et al., 2006; Heinzel et al., 2008; Lyon et al., 2009). In rapid pacing-induced failing dog atria, reduced T-tubular abundance was also found to be linked to altered subcellular Ca dynamics and AF development (Yeh et al., 2008; Dibb et al., 2009; Lenaerts et al., 2009). While accumulating evidence suggests that atrial T-tubular structure is present in most mammalian species, further investigations are clearly needed to understand whether there is remodeling in the failing and aged heart and its functional role in atrial SR Ca handling and AF development.

It is known that the cardiac Ca current during the normal AP contributes to the AP plateau and is involved in myocyte contraction. The voltage-gated L-type Ca channels (I_Ca_) are activated by membrane depolarization that leads to a small amount of inward Ca flux (I_Ca_) (Rougier et al., 1969). Ca entry via Ca current (I_Ca_) along with a much smaller amount of Ca influx via Na-Ca exchange (NCX) activates large quantities of Ca release from SR via ryanodine receptor channels (RyR; also called Ca triggered SR Ca release channels). This Ca triggered SR Ca release involves a transient increase in intracellular Ca [Ca]_i_ that initiates myocyte contraction as free Ca binds to the myofilaments (Bers, 2000). During the relaxation phase of the cells, intracellular free Ca ions will be removed from cytosol via: (1) pumping back to SR via a Ca pump SERCA2 (SR Ca-ATPase); (2) expulsion from the cell by NCXs; and (3) uptake by mitochondria via mitochondrial Ca uniporters (Bers, 2000).

Compared to ventricular myocytes, atrial myocytes have smaller Ca transient amplitude and a higher rate of intracellular Ca decay. This is due to an increased SERCA uptake and enhanced function of NCX to remove cytosolic Ca during the diastolic phase (Walden et al., 2009). The increased SERCA-dependent intracellular Ca removal is attributed to the greater amount of SERCA2 and less expression of SERCA inhibitory protein phospholamban (PLB) (Freestone et al., 2000; Walden et al., 2009). Another important feature of atrial myocytes is that atrial SR Ca content is greater than that of ventricular myocytes (Walden et al., 2009). With the greater atrial SR Ca content, atrial myocytes are prone to spontaneous diastolic SR Ca release when RyR channels are sensitized under pathological conditions (Venetucci et al., 2008; Bers, 2014).

We and others have previously discovered that increased diastolic SR Ca release causes abnormal ectopic activities, which lead to ventricular arrhythmogenesis in the failing heart (Ai et al., 2005; Yeh et al., 2008; Respress et al., 2012). During the diastolic phase, SR Ca release normally shuts off almost completely (~99%). However, increased diastolic RyR Ca release could be responsible for increased diastolic SR Ca leak and reduced systolic [Ca]_FR_ for a given L-type voltage-gated Ca current (I_ca_) as the release trigger (Bassani et al., 1995; Shannon et al., 2000; Bers, 2014). The increased diastolic SR Ca leakage along with an impaired function of Ca uptake due to altered SERCA2 elevates the amount of [Ca]_i_ and prolongs the [Ca]_i_ decay phase in HF (Bers, 2000, 2014). Then, increased Na influx via NCX for [Ca]_i_ removal can produce abnormal triggered activities (e.g., DADs) and initiate atrial arrhythmias (Bers, 2000, 2014). Studies suggest that alterations of Ca handling proteins including RyR2, PLB, and Cav1.2 contribute to changed intracellular Ca transients and diastolic SR Ca release (Schulman et al., 1992; DeSantiago et al., 2002; Wu et al., 1999). Others and we have previously demonstrated that activated CaMKII, a pro-arrhythmic signaling molecule, is critically involved in phosphorylation of RyR2-2815 and PLB-Thr17 (RyR2815-P, PLB17-P), which results in sensitized RyR channels that in turn leads to triggered activities and arrhythmia initiation due to diastolic SR Ca leak in pathologically altered ventricles (Hoch et al., 1999; Maier et al., 2003; Zhang et al., 2003; Ai et al., 2005; Yeh et al., 2008; Greiser et al., 2009; Sossalla et al., 2010; Respress et al., 2012). Recent studies indicate that alterations of CaMKII-dependent RyR phosphorylation are also exhibited in the atrium of chronic AF patients (Chelu et al., 2009; Neef et al., 2010). Results from several animal models have shown that these altered SR Ca handling proteins contribute to enhanced SR Ca leak and AF development (Chelu et al., 2009; Chiang et al., 2014). Although alteration of I_Ca_ could also contribute to abnormal SR Ca release, studies indicate that reduced I_Ca_ is a hallmark of AF induced electrical remodeling (Van Wagoner et al., 1999; Christ et al., 2004). CaMKII inhibition has been shown to improve the function of L-type Ca channel in mouse ventricular myocytes and cultured HL-1 atrial myocytes, which could be due to up-regulated expression of L-type Ca channel proteins (Zhang et al., 2005; Ronkainen et al., 2011). These results indicate that abnormal diastolic RyR Ca release could be the major cause of abnormal Ca handling in HF and chronic AF (Ai et al., 2005; Yeh et al., 2008 and Respress et al., 2012). However, other studies have reported inconsistent results of increased, reduced, or unchanged I_ca_ preceding the onset of AF in postoperative patients compared to that of patients at low risk for AF (Van Wagoner et al., 1999; Christ et al., 2004; Dinanian et al., 2008; Workman et al., 2009). Thus, the underlying mechanisms of abnormal Ca handling in AF onset and maintenance in the pathologically altered heart require further investigation.

In addition to altered phosphorylation of Ca handling proteins regulated by kinases, some protein phosphatases (PP1, PP2A) have also been found to play roles in regulating the phosphorylation state of channel proteins in failing ventricular myocytes (Ai et al., 2005, 2011; Ai and Pogwizd, 2005). However, contradictory results of the expression and activity of protein phosphatases have been reported in humans and animal models with chronic AF or paroxysmal AF (Christ et al., 2004; Chelu et al., 2009; Heijman et al., 2013; Voigt et al., 2014). It is clear that the functional role of protein phosphatases in atrial Ca handling and AF genesis need to be further explored.

## Stress signaling pathways in abnormal SR Ca handling and AF development in the failing or aged heart

**Figure 2:**
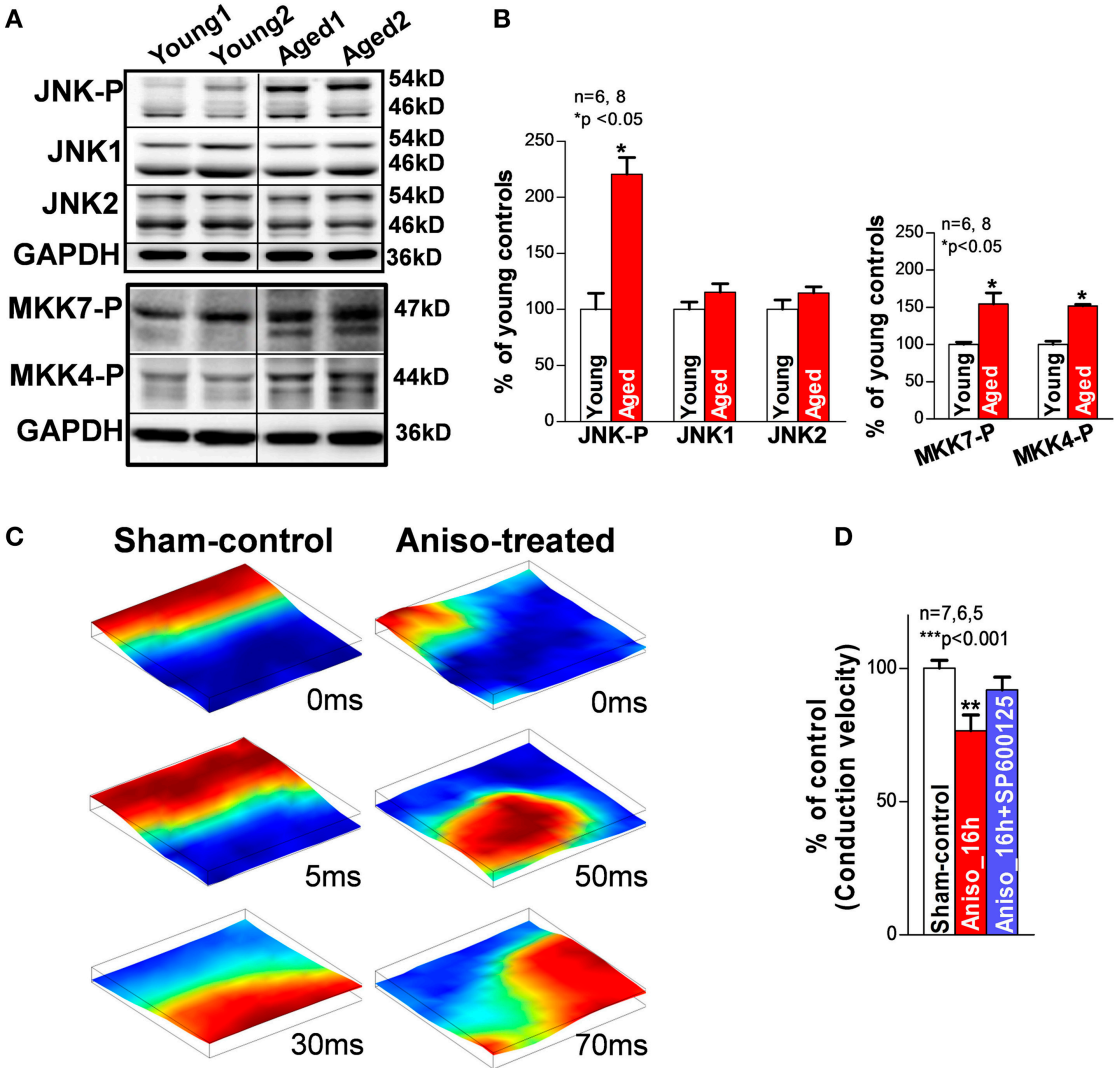
**(A,B)** Immunoblotting images and summarized data of phosphorylated JNK (JNK-P), JNK1, JNK2, and phosphorylated MKK7 and MKK4 (MKK7-P, MKK4-P) in young and aged rabbit LA. **(C)** Representative sequential images of uniformly propagated action potentials (CL = 200 ms) in sham-control HL-1 monolayers and broken and reentrant AP wave in an anisomycin-treated (24 h) monolayer. **(D)** Summarized data of conduction velocity between the three groups (^**^p < 0.01 vs. sham-control). This figure is modified from Yan et al. (2013).

It has been shown that failing and aged hearts exhibit increased intrinsic stress and higher susceptibility to extrinsic stress stimuli (Belmin et al., 1995; Beckman and Ames, 1998; Juhaszova et al., 2005; Li et al., 2005a; Yang et al., 2005; Judge and Leeuwenburgh, 2007; Neuman et al., 2007; He et al., 2011; Ismahil et al., 2014). JNK, a family member of the MAPKs, was discovered by Davis in the early of 90s (Davis, 2000). And then JNK was found to be activated in response to stress challenges to regulate cell proliferation, differentiation, apoptosis, cell survival, cell mobility and cytokine production (Davis, 2000; Bogoyevitch and Kobe, 2006; Raman et al., 2007). It is known that the JNK signaling pathway is critical in the development of cancer, diabetes, and cardiovascular diseases (CVD; e.g., HF, myocardial infarction, atherosclerosis) (Davis, 2000; Karin and Gallagher, 2005; Rose et al., 2010). Emerging evidence suggests that enhanced JNK activation is also linked to significantly elevated intrinsic stress (e.g., oxidative stress or inflammatory stress) (Liu et al., 2014; Sun et al., 2014). Studies have shown that rapid transient JNK activation appears in cultured myocytes and animals that are subjected to exercise or severe pressure overload, Boluyt et al. (2003), Nadruz et al. (2004, 2005) and Pan et al. (2005) while 24 h mechanically stretched myocytes or exercise trained animals showed reduced or unchanged JNK activity (Boluyt et al., 2003; Miyamoto et al., 2004; Roussel et al., 2008). These results indicate that JNK activation could be a dynamic response to the stress stimuli. Our laboratory recently discovered and reported for the first time (Yan et al., 2013) that activated JNK plays an important role in reduced gap junction channels and slowed conduction (Figure 2) that is associated with markedly increased pacing-induced AF *in vivo* in aged rabbits. Young rabbits subjected to a JNK activator (anisomycin) (Hazzalin et al., 1998; Petrich et al., 2004) challenge *in vivo* also exhibited dramatically increased incidence and duration of pacing-induced AT/AF, which is comparable to that found in aged hearts (Figure 1). While a significantly increased propensity for AF in aged humans has been well-recorganized, Benjamin et al. (1994), Go et al. (2001) and Rich (2009) our recent observations (Wu et al., 2014) suggest an increase in activated JNK in aging human atrium from healthy donor hearts (which were rejected for heart transplant due to technical reasons). Moreover, we demonstrated that JNK-induced gap junction remodeling impairs atrial conduction and causes formation of reentrant circuits in cultured atrial myocytes (Figures 2C,D) (Yan et al., 2013). However, previous studies suggest that gap junction remodeling is most likely to contribute to stabilization and maintenance of AF (Elvan et al., 1997; van der Velden et al., 1998, 2000; Dupont et al., 2001; Polontchouk et al., 2001; Kostin et al., 2002; Nao et al., 2003; Kanagaratnam et al., 2004; Sakabe et al., 2004; Wetzel et al., 2005; Nattel et al., 2008). Therefore, other mechanisms such as SR Ca handling dysfunction could be responsible for the initiation of atrial arrhythmias in aged hearts. To date, the role of JNK in SR Ca handling and AF development in the failing and aged heart remains unknown. Our recent results suggest that activated JNK plays an important role in SR Ca leak and AF development in aged animals as well as young animals with manipulated JNK activity. A computer simulation study (Xie et al., 2010) suggested that generating an ectopic beat in heart tissue with poorly coupled neighboring myocytes (slowed AP conduction) requires much fewer EAD or DAD-producing myocytes than in normal tissue composed of well-coupled cells. In another words, impaired intercellular coupling could make cardiac tissue more vulnerable for generating ectopic triggers that may initiate arrhythmias. Therefore, JNK-induced slowed conduction in the aged atria may create a favorable environment for JNK-induced abnormal Ca activities to form ectopic beats and even to initiate AF. Many questions regarding the underlying mechanisms of JNK-induced AF genesis remain unanswered. Further investigations are clearly needed in this important research area.

**Figure 3:**
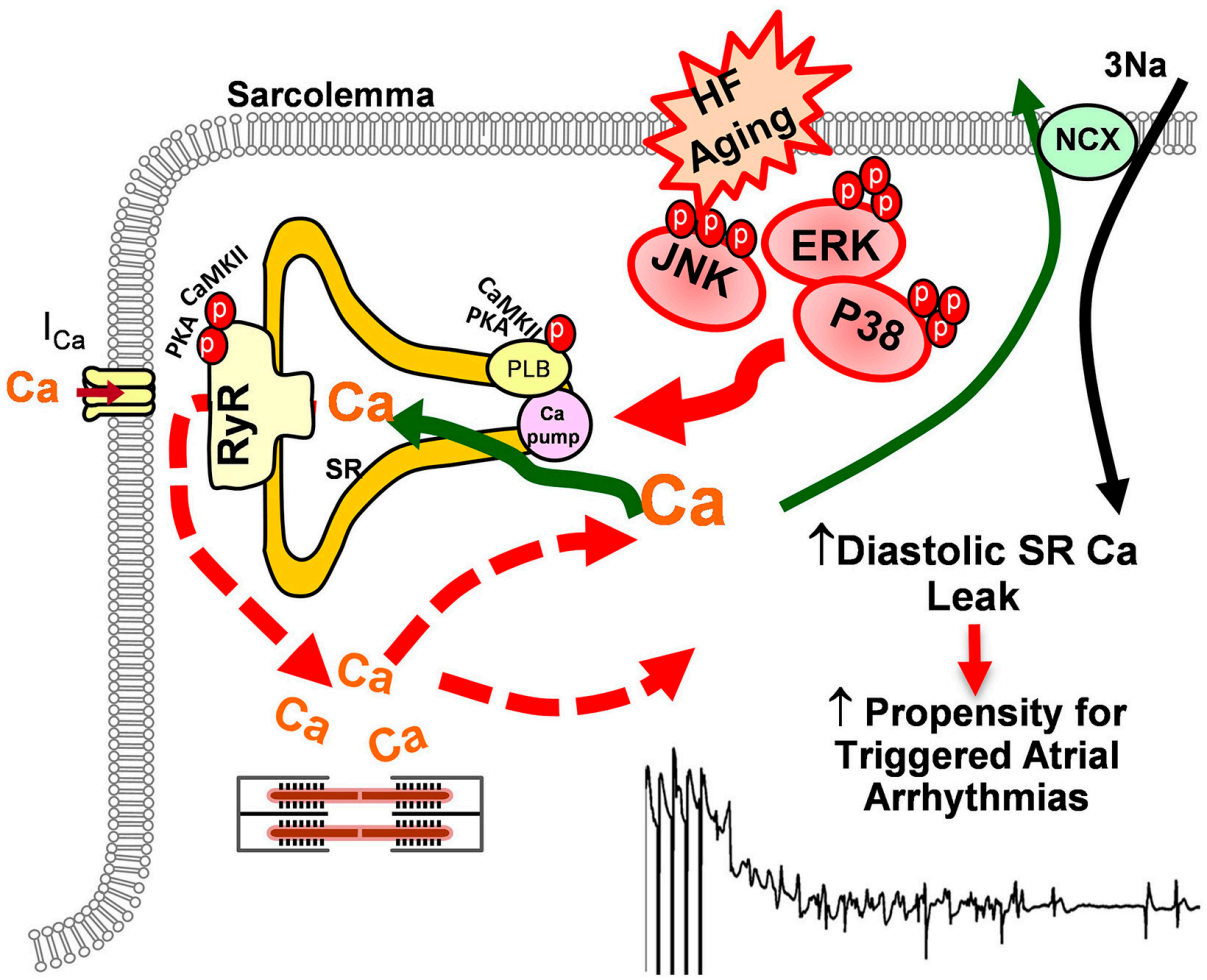
**Schematic outline of the potential impact of stress-response MAP kinases on sarcolemma reticulum (SR) Ca handling that may increase AF propensity in failing and/or aged atria**.

ERKs and p38 MAPKs are the other two important stress-response signaling pathways in cellular biology (Ramos, 2008; Rose et al., 2010). At the cellular level, the two stress signaling pathways modulate cell proliferation and differentiation, cytokinesis, transcription, cell death, and cell adhesion. Like JNK, both ERK and p38 are involved in various pathologies such as cardiovascular diseases, diabetes, and cancers (Davis, 2000; Kyriakis and Avruch, 2001; Karin and Gallagher, 2005; Kyoi et al., 2006; Yoon and Seger, 2006; Rose et al., 2010). While enhanced activity of ERK or p38 alone may or may not be required or sufficient for facilitating cardiac hypertrophy, both ERK and p38 were found to be activated in HF and these activated stress kinases are involved in pathological remodeling and AF development in the failing heart (Zechner et al., 1997; Wang et al., 1998; Li et al., 2001, 2005b; Cardin et al., 2003; Nishida et al., 2004; Purcell et al., 2007). Studies suggest that hypertrophic stimuli lead to an increase in L-type Ca transients and down-regulation of SERCA2 expression via activated ERK (Takahashi et al., 2004; Hagiwara et al., 2007; Huang et al., 2014). Ras, a GTPase, is able to activate ERK through a Ras-Raf-MEK cascade (Avruch et al., 2001). Ras signaling activated ERK was found to contribute to down-regulation of L-type Ca channels and reduced channel activity along with reduced SERCA2 protein expression in cultured myocytes (Ho et al., 1998, 2001; Huang et al., 2014). It was also found that Ras-ERK-modulated molecular remodeling led to decreased intracellular Ca transients and impaired SR Ca uptake, which could lead to enhanced arrhythmogenicity (Zheng et al., 2004). Moreover, recent work reported by Scharf et al. (2013) suggests that p38 directly regulates SERCA2 mRNA and protein expression via transcription factors Egr-1 and SP1. Taken together, emerging evidence indicates that the stress-response MAP kinases signaling cascades could be involved in cardiac Ca handling and AF development (Figure 3). However, more work needs to be done to further understand the underlying molecular and electrophysiological mechanisms of altered stress signaling cascades and their crosstalking in AF development in the failing and aged heart.

## Conclusion

Accumulating evidence suggests that abnormal SR Ca handling is associated with the initiation and development of AF. However, much work still needs to be done to further uncover the underlying molecular and electrophysiological mechanisms of AF initiation and maintenance in diseased and aged hearts. To date, most of the mechanistic studies of SR Ca dynamics have been performed in isolated myocytes. However, isolated myocytes provide limited information regarding the spatial complexity of SR Ca kinetics in the 3-dimensional myocardial structure, which is completely disrupted by the enzymatic dissociation procedure of cell isolation. Thus, measuring Ca dynamics in intact atria using high-resolution Ca imaging should be considered in future studies to obtain important information about the relationship of SR Ca handling and APs, as well as their role in arrhythmogenesis. At present, emerging evidence indicates a link between altered stress signaling cascades and abnormal Ca handling in pathologically altered atrium. Further understanding of the underlying mechanisms of stress-induced AF development in the failing and/or aged heart could reveal potential effective therapeutic strategies for AF prevention and treatment.

### Conflict of interest statement

The author declares that the research was conducted in the absence of any commercial or financial relationships that could be construed as a potential conflict of interest.
